# H5N1 infection impairs the alveolar epithelial barrier through intercellular junction proteins via Itch-mediated proteasomal degradation

**DOI:** 10.1038/s42003-022-03131-3

**Published:** 2022-03-01

**Authors:** Tao Ruan, Yuling Sun, Jingting Zhang, Jing Sun, Wei Liu, Richard A. Prinz, Daxin Peng, Xiufan Liu, Xiulong Xu

**Affiliations:** 1 College of Veterinary Medicine, Yangzhou University, Yangzhou, 225009 Jiangsu Province China; 2 Institute of Comparative Medicine, Yangzhou University, Yangzhou, 225009 Jiangsu Province China; 3 Department of Surgery, NorthShore University Health System, Evanston, IL 60201 USA; 4 Animal Infectious Disease Laboratory, College of Veterinary Medicine, Yangzhou University, Yangzhou, Jiangsu Province 225009 China; 5 Jiangsu Co-innovation Center for Prevention and Control of Important Animal Infectious Diseases and Zoonosis, Yangzhou University, Yangzhou, 225009 Jiangsu Province China

**Keywords:** Mechanisms of disease, Influenza virus

## Abstract

The H5N1 subtype of the avian influenza virus causes sporadic but fatal infections in humans. H5N1 virus infection leads to the disruption of the alveolar epithelial barrier, a pathologic change that often progresses into acute respiratory distress syndrome (ARDS) and pneumonia. The mechanisms underlying this remain poorly understood. Here we report that H5N1 viruses downregulate the expression of intercellular junction proteins (E-cadherin, occludin, claudin-1, and ZO-1) in several cell lines and the lungs of H5N1 virus-infected mice. H5N1 virus infection activates TGF-β-activated kinase 1 (TAK1), which then activates p38 and ERK to induce E3 ubiquitin ligase Itch expression and to promote occludin ubiquitination and degradation. Inhibition of the TAK1-Itch pathway restores the intercellular junction structure and function in vitro and in the lungs of H5N1 virus-infected mice. Our study suggests that H5N1 virus infection impairs the alveolar epithelial barrier by downregulating the expression of intercellular junction proteins at the posttranslational level.

## Abstract (web summary)

Ruan et al. investigate the effect of H5N1 infection on the expression of intercellular junction proteins (E-cadherin, Occludin, Claudin-1, and ZO-1), which they find results in reduced expression of these proteins via activation of TAK1, p38, and ERK, leading to an induction of Itch. The authors report that Itch activation then results in ubiquitination and degradation of these cellular junction proteins.

## Introduction

The composition of the alveolar epithelium is 95% flat-shaped type I epithelial cells and 5% cubic-like type II epithelial cells^1,2^. This epithelial monolayer functions as a barrier to prevent the passage of proteins and other macromolecules from the alveolar capillary and microvessels into the alveolar space^1^. The alveolar epithelium exerts its barrier function largely through the intercellular junction structure: the tight and adherens junctions. The tight junction is a homodimeric structure formed by the interaction of the extracellular ring structure of occludin or the member of the claudin family, both of which are the four-pass transmembrane proteins^3–6^. The adherens junction is formed through the interaction of five IgG-like loops in the extracellular domain of E-cadherin or Nectin proteins. The intracellular C-terminal domain of E-cadherin and Nectin is engaged with β-catenin. ZO-1 is an adapter protein that interacts with the cytoplasmic regions of the tight junction and adherens junction proteins to form filaments, which stabilize the intercellular junction structure^3–5^.

Claudins and occludin are the major components of the tight junction in epithelial and endothelial cells. Their expression is tightly regulated at both transcriptional and posttranslational levels^7,8^. Of the total 16 claudins, claudin-1, -2, -4, -5, -8, -16 can be ubiquitinated by LNX1p80, an E3 ubiquitin ligase, and degraded through the proteasomal pathway^7,8^. Occludin is ubiquitinated by Itch, a HECT domain-containing E3 ubiquitin ligase, and undergoes proteasomal degradation^7–9^. Occludin ubiquitination occurs in the intestinal epithelial cells in patients with irritable bowel syndrome, in the tight junction of the Sertoli cells during spermatogenesis, and in the blood-brain barrier with brain ischemia and ischemia-reperfusion injury^7,8^. E-cadherin, an adherens junction protein, is ubiquitinated by several E3 ubiquitin ligases including Hakai, Fbxl20, and MARCH8^10^. Ubiquitinated E-cadherin undergoes endocytosis in endothelial cells^10^ and proteasomal degradation in epithelial cells^11^. ZO-1 is ubiquitinated by interleukin-6 (IL-6)-activated Ubr-1 (Ub ligase E3 component n-recognin-1) in Japanese encephalitis viral (JEV)-infected astrocytes, leading to ZO-1 degradation and disruption of the blood-brain barrier^12,13^.

Influenza A virus (IAV) is a common respiratory tract pathogen. Influenza often presents as seasonal epidemics and causes hundreds of thousands of deaths annually^14^. The prevalent subtypes of IAV that infect humans are the H1N1 and H3N2 viruses. The 2009 influenza pandemic caused 150 to 600 thousand deaths^15,16^. H5N1 and its derivative subtypes (H5NX) are highly pathogenic IAV of avian origin^17^. Since it was first isolated in 1996 from diseased geese in Southern China, H5N1 viruses have spread to more than 70 countries. In addition to inflicting heavy losses to the poultry industry^18,19^, H5N1 viruses cause sporadic human infections^17,20^. A total of 861 confirmed human cases with 455 deaths have been reported in 17 countries since 1997. H5N6 viruses, the second H5 subtype that infects humans^21^, has replaced H5N1 strains as the dominant epidemic subtype in poultry^22,23^. By March 2020, 24 H5N6-infected human infections with eight deaths have been reported^24^. H5N6 viruses have a broad host range including poultry, waterfowl, wild birds, and some mammals such as pigs and cats^25,26^. H5N1 and H5Nx viruses pose a major public health threat^24^.

Immunocompromised individuals succumbed to influenza A virus infections often develop acute respiratory distress syndrome (ARDS). Its pathogenesis is characterized by disintegration of alveolar epithelial and endothelial barriers and increased alveolar permeability^27–29^. Impaired barriers result in alveolar protein-rich effusion, edema, and infiltration with red blood cells and inflammatory cells. Alveoli filled with effusion and edema lose their gas exchange function leading to dyspnea^27,29^. Patients with the ARDS often progress into other serious conditions such as viral pneumonia, secondary bacterial infections, and multiple organ failure that account for a death rate of approximately 60%^30,31^. Local inflammation in lung tissue can increase the permeability of the vascular endothelial barrier^3,4,27,28^, whereas programmed death of alveolar epithelial cells may also disrupt the natural barrier of alveolar epithelium, leading to increased alveolar permeability^27^.

We recently reported that H1N1 viruses suppress the expression of the intercellular junction proteins of the alveolar barrier at the transcriptional level by activating the Gli1 transcription factor^32^. Our present study focuses on the posttranslational modification and downregulation of intercellular junction proteins in IAV-infected cells and their pathological importance. Here we report that H5N1 viruses damaged the intercellular junction structure by accelerating the turnover of several junction proteins. Further investigation revealed that H5N1 viruses activated TAK1 and its two downstream MAP kinases, p38 and ERK, leading to increased Itch expression and ubiquitination of occludin and potentially other junction proteins. Our study provides mechanistic insights into how H5N1 viruses disrupt the alveolar barrier and advances our understanding of the pathogenesis of the H5N1 virus.

## Results

### H5N1 viruses down-regulate the expression of Gli1, Snail, and intercellular junction proteins

**Fig. 1:**
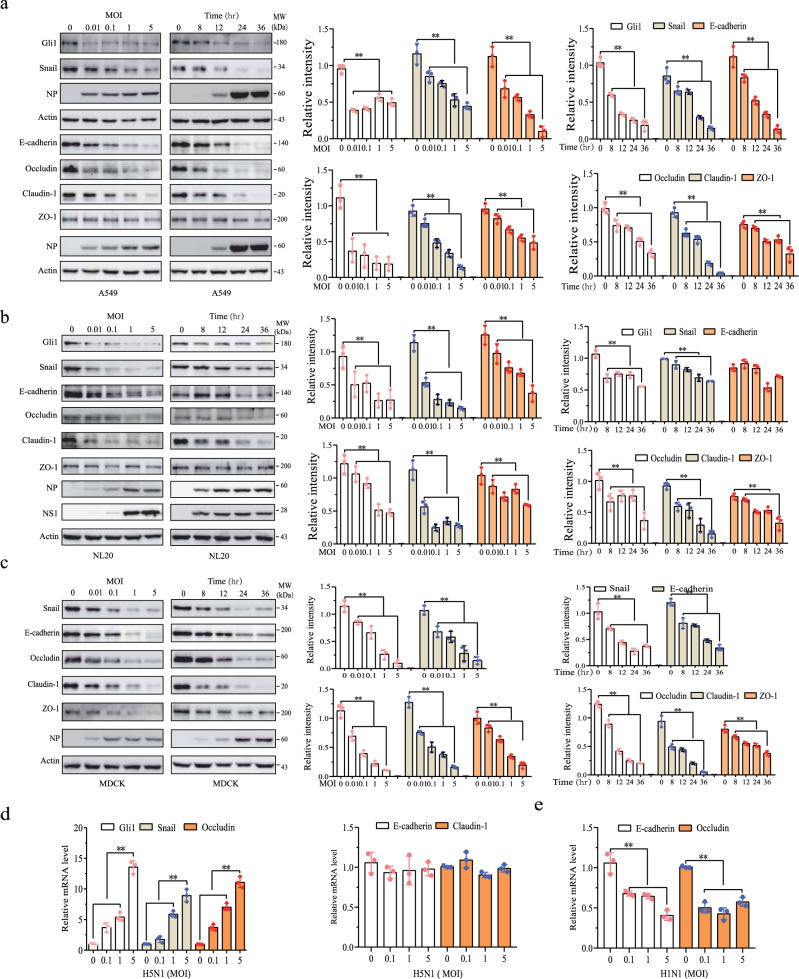
The H5N1 virus down-regulates the levels of Gli1, Snail, and junction proteins. A549 (**a**), NL20 (**b**), and MDCK (**c**) cells were infected with various MOI of H5N1 viruses (the SY strain) for 24 h or with H5N1 viruses (1 MOI) for the indicated lengths of time. Cell lysates were analyzed for the levels of Gli1, Snail, and four junction proteins by Western blot. The density of the bands was analyzed by using NIH Image-J software and normalized by the arbitrary units of β-actin. Data are the mean ± SD of three experiments. *p < 0.05, **p < 0.01, com*p*ared to uninfected control. **d**, **e** The effect of influenza viruses on *GLI1*, *SNAI*, *CDH*, CLDN1, and *OCLN* mRNA expression. Total mRNA from A549 cells infected with the indicated MOI of H5N1 (**d**) or H1N1 (**e**) viruses was analyzed for *GLI1*, *SNAI*, *OCLN, CLDN1, and CDH* mRNA levels by RT-PCR. Data are the mean ± SD of three independent experiments. **p < 0.01, compared to uninfected control. *p < 0.05, **p < 0.01.

We recently reported that H1N1 viruses activate the PI-3 and MAP kinase pathways to induce the expression of Gli1 and Snail, two transcription factors that play an important role in down-regulating the expression of intercellular junction proteins at the transcriptional levels^32^. Unlike H1N1, H5N1 viruses do not activate the PI-3 kinase pathway^33^. We first investigated if H5N1 viruses could also induce the expression of Gli1 and Snail, subsequently leading to the suppression of intercellular junction protein expression. Unexpectedly, H5N1 viruses did not increase but rather decreased the levels of Gli1 and Snail in a dose- and time-dependent manner in A549 (a human lung cancer cell line), NL20 (a human noncancerous alveolar epithelial cell line), and MDCK cells (a non-cancerous canine kidney cell line widely used for influenza virus study and for epithelial-to-mesenchymal transition) (Fig. 1a–c). However, H5N1 viruses still effectively lowered the levels of E-cadherin, occludin, and claudin-1 expression in a dose- and time-dependent manner in all three cell lines (Fig. 1a–c). H5N1 viruses down-regulated ZO-1 expression at much lower magnitudes than the other three junction proteins in these three cell lines (Fig. 1a–c). In addition, the levels of Gli1, Snail, and these four junction proteins were also decreased in A549 cells infected with a second strain of the H5N1 virus (CK10) and an H5N6 strain (Y6) (Supplementary Fig. 1).

We next determined if H5N1 viruses downregulated the expression of Gli1, Snail, and intercellular junction proteins at the transcriptional level. As shown in Fig. 1d, H5N1 viruses increased Gli1, Snail, and occludin mRNA levels but did not affect the E-cadherin and claudin-1 mRNA levels in A549 cells. We reported earlier that the H1N1 virus activates the Gli1 promoter and induces Gli1 mRNA expression^32^. Here we show that H1N1 viruses did decrease the levels of E-cadherin and Occludin levels (Fig. 1e), probably from transcriptional repression by Snail since its expression is increased in H1N1 virus-infected cells^32^. Nevertheless, these observations suggest that H5N1 viruses suppress the expression of Gli1, Snail, and four junction proteins at the posttranslational level.

### H5N1 viruses downregulate intercellular junction proteins by ubiquitin-mediated proteasomal degradation

**Fig. 2:**
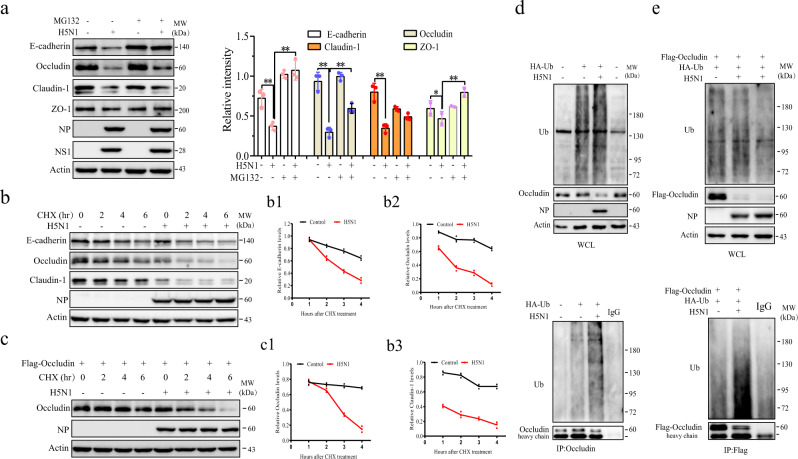
H5N1 virus induces E-cadherin, occludin, and claudin-1 degradation. **a** H5N1 viruses accelerate the turnover of intercellular junction proteins. A549 cells infected with 1 MOI of H5N1 viruses were incubated for 24 h in the absence or presence of MG132 (10 μM). Cell lysates were analyzed by Western blot for the levels of E-cadherin, occludin, claudin-1, ZO-1, and the viral NP and NS1 proteins with their specific antibodies. **b** H5N1 viruses shorten the half-life of intercellular junction proteins. A549 cells were left uninfected or infected with H5N1 viruses (1 MOI). After incubation for 12 h, CHX (10 μM) was added and incubated for the indicated lengths of time. Cell lysates were prepared and analyzed for E-cadherin, occludin, claudin-1, and NP by Western blot. **c** Quantification of relative E-cadherin levels (from **b**). **d** Quantification of relative Occludin levels (from **b**). **e** H5N1 viruses shorten the half-life of occludin. A549 cells were transiently transfected with the pcDNA3.1 expression vector encoding the FLAG-occludin gene. After incubation for 24 h, the cells were then left uninfected or infected with the H5N1 virus (1 MOI). After incubation for 12 h, CHX (10 μM) was added and incubated for the indicated lengths of time. Cells were harvested and analyzed for occludin by Western blot. The density of bands was analyzed using NIH Image-J software and normalized by the arbitrary units of β-actin. **f** Quantification of relative Occludin levels (from **e**). **g** Quantification of relative Claudin-I levels (from **b**). Data presented in **c**, **d**, **f**, and **g** are the mean ± SD from one of three experiments in triplicate. **h**, **i** H5N1 virus induces occludin ubiquitination. A549 cells were transiently transfected with an HA-ubiquitin expression vector alone (**h**) or plus the pcDNA3.1 expression vector encoding the FLAG-occludin gene (**i**). After incubation for 24 h, the cells were left uninfected or infected with the H5N1 virus (1 MOI). After incubation for another 24 h, cell lysates were prepared and immunoprecipitated with an antibody against occludin (**h**) or FLAG (**i**). Whole-cell lysates (WCL) and immunoprecipitates were analyzed with antibodies against ubiquitin, NP, occludin, and β-actin. The data represent one of three independent experiments. *p < 0.05, **p < 0.01.

In addition to being transcriptionally regulated, occludin and other junction proteins could be ubiquitinated for proteasomal degradation^7,8^. Here we tested if MG132, a proteasomal inhibitor, could block H5N1 virus-induced junction protein downregulation. As shown in Fig. 2a, MG132 (5 μM) did not significantly alter the levels of E-cadherin, occludin, claudin-1, and ZO-1 in uninfected A549 cells but largely restored the levels of these junction proteins in H5N1 virus-infected cells. We next measured the half-life of E-cadherin, occludin, and claudin-1 in uninfected and H5N1 virus-infected A549 cells in the presence of cycloheximide (CHX) (10 μM). As shown in Fig. 2b, the half-life of E-cadherin and occludin in uninfected cells was > 6 h in uninfected cells. H5N1 virus infection shortened the half-life of E-cadherin (Fig. 2c) and occludin (Fig. 2d) to approximately 3 h. While H5N1 viruses dramatically lowered the levels of claudin-1, they did not significantly decrease its half-life, probably due to the levels of claudin-1 already being low when CHX was added. (Fig. 2b, g). The effect of H5N1 viruses on the half-life of occludin was further investigated in A549 cells overexpressing occludin. As shown in Fig. 2e, f, H5N1 viruses accelerated the degradation of occludin in A549 cells transiently transfected with an expression vector encoding the FLAG-occludin gene.

We next tested if down-regulation of occludin in H5N1 virus-infected A549 cells was indeed due to its ubiquitination. A549 cells were left untransfected or transfected with an expression vector encoding the HA-ubiquitin gene (Fig. 2h) or with the HA-ubiquitin expression vector plus the FLAG-occludin expression vector (Fig. 2i). After incubation for 24 hr, the cells were left uninfected or infected with H5N1 viruses and incubated for another 24 h. As shown in Fig. 2h, i (top panels), there was no significant difference in the levels of total protein ubiquitination in the cell lysates of uninfected or H5N1 virus-infected A549 cells. However, immunoprecipitation with an antibody against occludin revealed that the H5N1 virus dramatically increased occludin ubiquitination in A549 cells transfected with the HA-ubiquitin vector or with the HA-ubiquitin expression vector plus the FLAG-occludin expression vector (Fig. 2h, i, bottom panels).

### H5N1 viruses induce Itch to ubiquitinate occludin

**Fig. 3:**
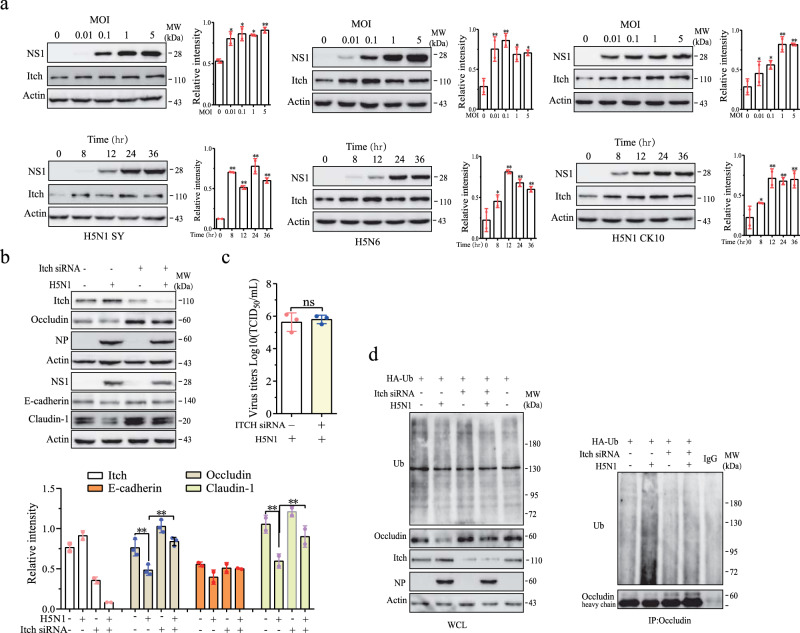
H5N1 viruses degrade intercellular junction proteins by inducing Itch expression. **a** H5N1 viruses induce Itch expression. A549 cells were infected with various MOI of two H5N1 strains (SY and CK10) and H5N6 viruses (the Y6 strain) for 24 hr or with these viruses (1 MOI) for the indicated lengths of time. Cell lysates were analyzed for the levels of Itch and actin by Western blot. **b** Itch knockdown blocks H5N1 virus-induced downregulation of junction proteins. A549 cells were transfected with a scrambled control or Itch siRNA. After incubation for 24 h, the cells were left uninfected or infected with H5N1 virus (1 MOI) and incubated for another 24 h. Cell lysates were analyzed for the levels of Itch and junction proteins by Western blot with the indicated antibodies. **c** The conditioned media from H5N1 virus-infected cells were analyzed for virus titers by measuring the TCID50 values. **d** Itch knockdown blocks H5N1 virus-induced occludin ubiquitination. A549 cells were transfected with a scrambled control or Itch siRNA plus the expression vector encoding HA-ubiquitin. After incubation for 24 h, the cells were left uninfected or infected with H5N1 virus (1 MOI) and incubated for another 24 h. Cell lysates were immunoprecipitated with an antibody against occludin. Normal mouse IgG was included as a negative control. Whole-cell lysates (WCL) and immunoprecipitates were analyzed with antibodies against ubiquitin, NP, occludin, and β-actin. The data represent one of three independent experiments.

Itch is an E3 ubiquitin ligase responsible for ubiquitinating occludin^34,35^. Here we tested if occludin was indeed ubiquitinated by Itch in H5N1 virus-infected A549 cells. We first determined the ability of H5N1 viruses to induce Itch expression. As shown in Fig. 3a, Itch expression was increased in A549 cells infected with two strains of the H5N1 virus (SY and CK10) or the H5N6 virus (Y6) largely in a dose- and time-dependent manner. Itch siRNA dramatically decreased Itch expression in A549 cells (Fig. 3b). Itch knockdown blocked H5N1 virus-induced downregulation of occludin, E-cadherin, and claudin-1 but did not significantly affect the expression of the NP and NS1 proteins of H5N1 viruses (Fig. 3b) nor the virus titers in the conditioned media of H5N1 virus-infected cells (Fig. 3c). Immunoprecipitation revealed that H5N1 viruses increased occludin ubiquitination in A549 transfected with the control siRNA but was unable to do so in the cells transfected with Itch siRNA (Fig. 3d). These observations suggest that Itch is the E3 ubiquitin ligase primarily responsible for H5N1 virus-induced ubiquitination.

### p38 and ERK but not JNK activation is required for H5N1 virus-induced Itch expression and junction protein degradation

**Fig. 4:**
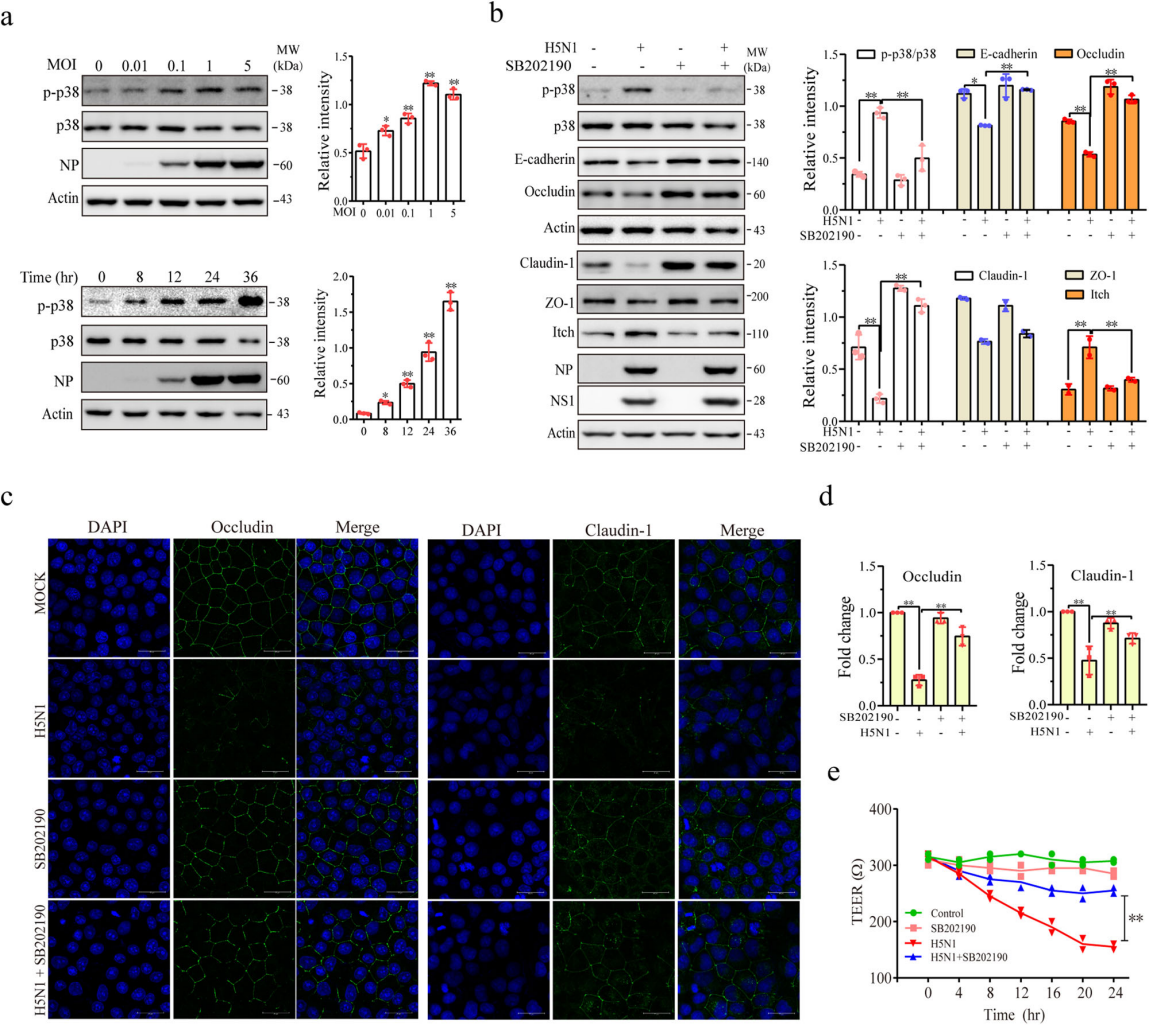
Role of p38 in H5N1 virus-induced Itch expression and degradation of junction proteins. **a** H5N1 viruses induce p38 phosphorylation. A549 cells were infected with the indicated MOI of H5N1 viruses for 24 h or infected with 1 MOI of H5N1 viruses for the indicated lengths of time. Cell lysates were analyzed for the levels of p38 phosphorylation, NP, and β-actin by Western blot. **b** SB202190 blocks H5N1 virus-induced Itch expression and junction protein downregulation. A549 cells were left uninfected or infected with the H5N1 virus (1 MOI). After incubation for 12 hr, SB202190 (10 μM) was added and incubated for another 12 h. Cell lysates were prepared and analyzed for the levels of p38 phosphorylation, Itch, and junction proteins by Western blot with the indicated antibodies. The density of bands was analyzed using NIH Image-J software and normalized by the arbitrary units of total p38 or β-actin. Data represent the mean ± SD of three experiments. *p < 0.05, **p < 0.01. **c** SB202190 blocks H5N1 virus-induced disruption of intercellular junction structure. A549 cells were left uninfected or infected with H5N1 viruses (1 MOI). The cells were then incubated in the absence or presence of SB202190 (10 μM) for 24 h. The cells were fixed and stained with the antibodies against occludin or claudin-1. **d** Quantification of fluorescence signals. The monolayers immunostained for occludin and claudin-1 were analyzed for immunofluorescence intensity by using Image J software. The fluorescent signals of intercellular junctions were quantified and plotted as bar graphs. Data represent the mean ± SD of five random fields (40X) from one of three independent experiments with similar results. **e** SB202190 prevents the H5N1 virus-induced decrease of electronic resistance. A549 cells seeded in the Transwell inserts were left uninfected or infected with H5N1 viruses in the absence or presence of SB202190 (10 μM). TEER was measured at the indicated times. The results represent the mean ± SD of three independent experiments. *p < 0.05, **p < 0.01.

p38 induces Itch expression to degrade occludin^36^. Here we determined if p38 played a role in H5N1 virus-induced Itch expression and the degradation of intercellular junction proteins. As shown in Fig. 4a, H5N1 viruses time-dependently increased p38 phosphorylation in A549 cells infected with 1 MOI of H5N1 viruses or dose-dependently increased p38 phosphorylation in A549 cells infected with H5N1 virus 24 hours post infection (hpi). SB202190, a p38 kinase-specific inhibitor, largely blocked H5N1 virus-induced p38 phosphorylation and restored the levels of E-cadherin, claudin-1, and occludin in H5N1 virus-infected A549 cells (Fig. 4b). SB202190 slightly increased the levels of these proteins in uninfected cells but did not affect the expression of the NP and NS1 proteins of H5N1 viruses (Fig. 4b). SB202190 blocked H5N1 virus-induced Itch expression (Fig. 4b). Immunofluorescence (IF) staining revealed that SB202190 partially restored the expression of occludin and claudin-1 on the cell membrane of H5N1 virus-infected A549 cells (Fig. 4c). IF intensity analysis revealed that H5N1 virus infection significantly decreased occludin and claudin-1 signals located in intercellular junctions. This decrease was largely reversed by SB202190 (Fig. 4d). The transepithelial electrical resistance (TEER) assay revealed that SB202190 moderately prevented the decrease of electric resistance in H5N1 virus-infected A549 cells (Fig. 4e). The effect of SB202190 on the permeability of the H5N1 virus-infected NL20 monolayer was not investigated due to the poor ability of H5N1 virus to decrease TEER in this cell line.

**Fig. 5:**
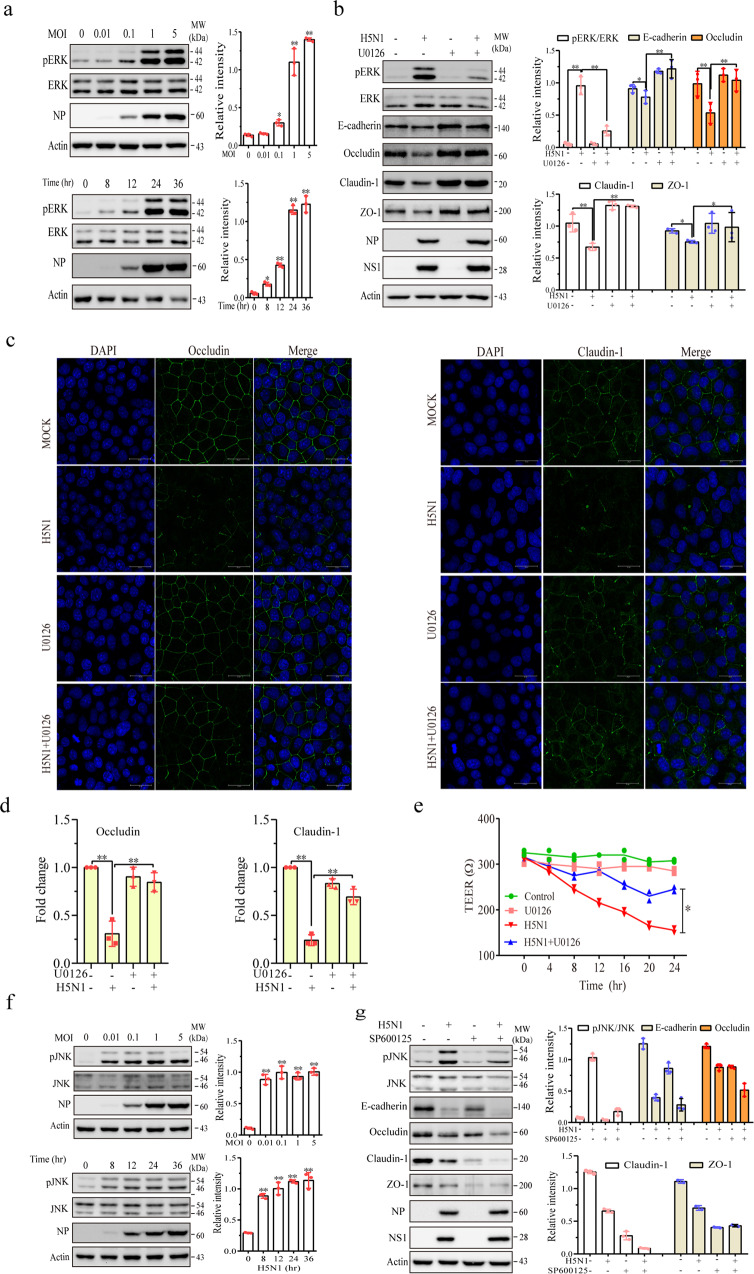
Role of ERK in H5N1 virus-induced Itch expression and the degradation of junction proteins. **a** H5N1 viruses induce ERK phosphorylation. A549 cells were infected with the indicated MOI of H5N1 viruses for 24 h or infected with H5N1 viruses (1 MOI) for the indicated lengths of time. ERK1/2 phosphorylation was analyzed by Western blot. **b** U0126 blocks H5N1 virus-induced Itch expression and junction protein downregulation. A549 cells were left uninfected or infected with H5N1 viruses (1 MOI). After incubation for 12 h, U0126 (10 μM) was added and incubated for another 12 h. Cell lysates were analyzed for the levels of ERK phosphorylation, Itch, and junction proteins by Western blot with the indicated antibodies. **c** U0126 blocks H5N1 virus-induced disruption of intercellular junction structure. A549 cells were infected with H5N1 viruses (1 MOI). After incubation for 12 h, the cells were incubated in the absence or presence of U0126 (10 mM) for another 12 h. The cells were fixed and then stained with antibodies against occludin and claudin-1. **d** Quantification of fluorescence signals. The monolayers immunostained for occludin and ZO-1 in **c** were analyzed for immunofluorescence intensity by using Image J software. The fluorescent signals of intercellular junction proteins were quantified and plotted as bar graphs. The data represent the mean ± SD of five random fields (40X) from one of three independent experiments with similar results. **e** U0126 blocks the H5N1 virus-induced decrease of electronic resistance. A549 cells seeded in Transwell inserts were left uninfected or infected with H5N1 viruses in the absence or presence of U0126 (10 μM). TEER was measured at the indicated lengths of time. The results represent the mean ± SD of three independent experiments. *p < 0.05, **p < 0.01. **f** H5N1 viruses induce JNK phosphorylation. A549 cells were infected with the indicated MOI of H5N1 viruses for 24 h or infected with H5N1 viruses (1 MOI) for the indicated lengths of time. JNK phosphorylation was analyzed by Western blot. **g** SP600125 does not block H5N1 virus-induced downregulation of junction proteins. A549 cells were left uninfected or infected with H5N1 viruses (1 MOI). After incubation for 12 h, U0126 (10 μM) was added and incubated for another 12 h. Cell lysates were analyzed for the levels of ERK phosphorylation, Itch, and junction proteins by Western blot with the indicated antibodies. The density of bands was analyzed using NIH Image-J software and normalized by the arbitrary units of ERK, JNK, or β-actin. Data are the mean ± SD of three experiments. *p < 0.05, **p < 0.01.

We next determined the effect of ERK on the levels of Itch and junction proteins. Again, H5N1 viruses dose- and time-dependently increased ERK phosphorylation in A549 cells (Fig. 5a). U0126, a MEK inhibitor, blocked H5N1 virus-induced ERK phosphorylation and restored E-cadherin, ZO-1, occludin, and claudin-1 levels in A549 cells (Fig. 5b). U0126 did not significantly change the levels of these proteins in uninfected cells (Fig. 5b). IF staining showed that U0126 restored the expression of occludin and claudin-1 on the cell membrane of H5N1 virus-infected A549 cells (Fig. 5c). Immunofluorescence intensity analysis revealed that IAV infection significantly decreased the occludin and claudin-1 signals located in the intercellular junctions, which was largely reversed by U0126 (Fig. 5d). The TEER assay revealed that inhibition of the MAP kinase pathway by U0126 moderately prevented the decrease of electric resistance in H5N1 virus-infected A549 cells (Fig. 5e).

We then determined if JNK activation also contributed to the H5N1 virus-induced downregulation of intercellular junction proteins. As shown in Fig. 5f, H5N1 viruses at a low MOI could rapidly induce JNK phosphorylation. SP600125, a JNK kinase inhibitor, largely inhibited H5N1 virus-induced JNK phosphorylation and did not restore but rather potentiated the downregulation of E-cadherin, claudin-1, occludin, and ZO-1 expression in H5N1 virus-infected A549 cells (Fig. 5g). SP600125 did not affect the expression of the viral NP and NS1 proteins of H5N1 viruses (Fig. 5g) since it was added 12 hr after inoculation.

### H5N1 viruses downregulate intercellular junction protein expression by activating TAK1

**Fig. 6:**
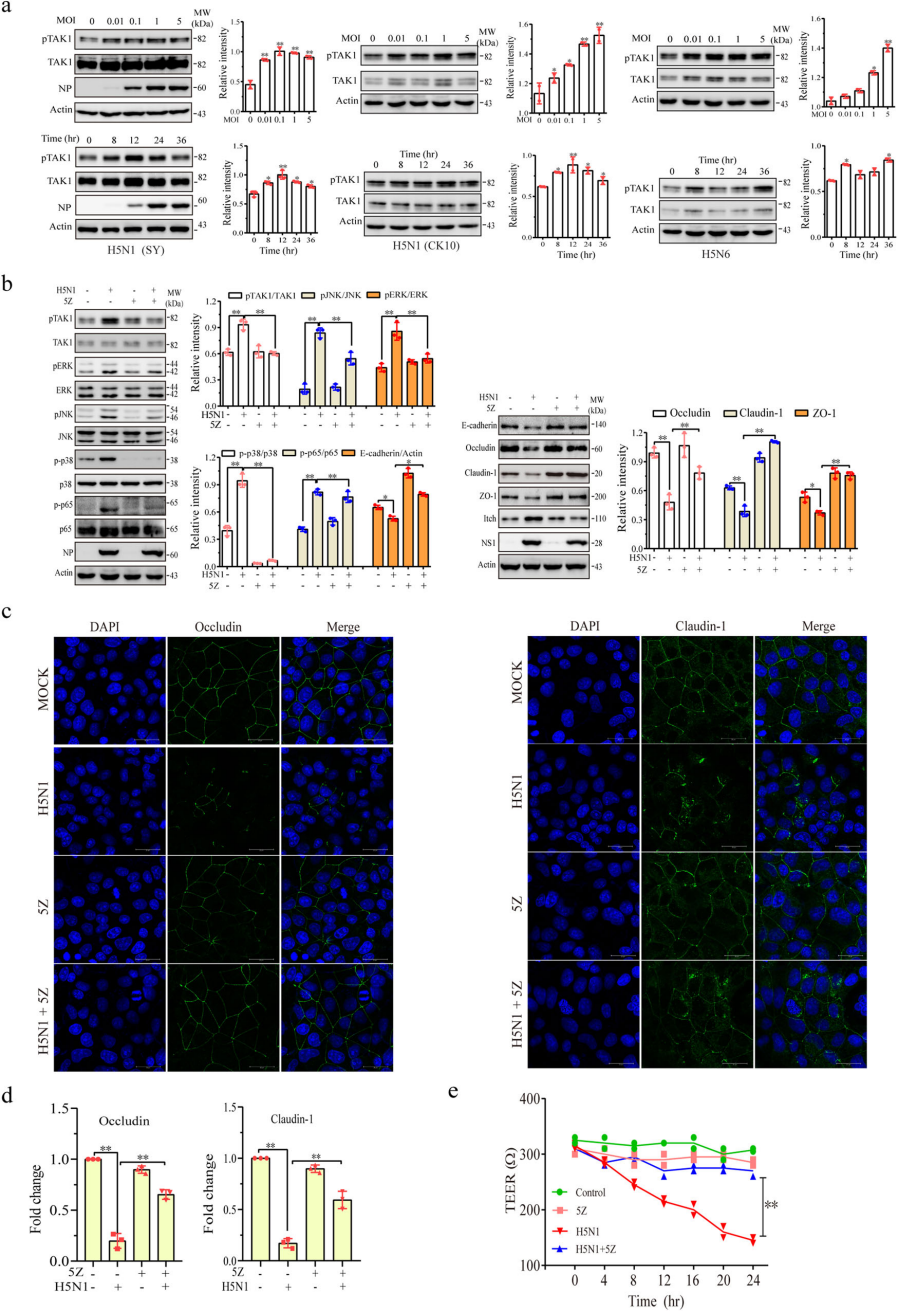
Role of TAK1 in degrading junction proteins. **a** H5N1 viruses induce TAK1 phosphorylation. A549, MDCK, and NL20 cells were infected with various MOI of the H5N1 viruses for 24 h or with the H5N1 viruses (1 MOI) for the indicated lengths of time. Cell lysates were analyzed for the levels of TAK1 phosphorylation, NP, NS1, and β-actin by Western blot. **b** 5Z blocks H5N1 virus-induced Itch expression and junction protein downregulation. A549 cells were left uninfected or infected with 1 MOI of H5N1 viruses. After incubation for 12 h, 5Z (5 μM) was added and then incubated for another 12 h. Cell lysates were analyzed by Western blot for TAK1, ERK, JNK, p38, and p65 phosphorylation and for the levels of junction proteins with their specific antibodies. The density of bands was analyzed using NIH Image-J software and normalized by the arbitrary units of β-actin. Data are the mean ± SD of three experiments. *p < 0.05, **p < 0.01. **c** 5Z blocks H5N1 virus-induced disruption of intercellular junction structure. A549 cells seeded on coverslips were treated as above. Cells were immunostained with antibodies against occludin and claudin-1 and visualized under a confocal microscope. **d** Quantification of fluorescence signals. The monolayers immunostained for occludin and claudin-1 in **c** were analyzed for immunofluorescence intensity by using Image J software. The fluorescent signals of intercellular junctions were quantified and plotted as bar graphs. Data represent the mean ± SD of five random fields (40X) from one of three independent experiments with similar results. **e** 5Z blocks the H5N1 virus-induced decrease of electronic resistance. A549 cells seeded in Transwell inserts were left uninfected or infected with H5N1 viruses. After incubation for 12 h, 5Z (5 μM) was added and then incubated for another 12 h. TEER was measured at the indicated times. The results represent the mean ± SD of three independent experiments. **p < 0.01.

TAK1 plays an important role in activating all three MAP kinase cascades^37,38^. TAK1 phosphorylation was increased in A549 cells infected with two strains of H5N1 viruses (SY, CK10) or with H5N6 viruses (Y6) largely in a dose-dependent manner (Fig. 6a). Infections with these viruses led to rapid induction of TAK1 phosphorylation (Fig. 6a). 5Z-7-oxozeaenol (5Z), a TAK1-specific inhibitor, blocked H5N1 virus-induced phosphorylation of TAK1, ERK, JNK, p38, and the p65 subunit of NK-κB. 5Z restored E-cadherin, claudin-1, occludin, ZO-1, and Itch protein levels in H5N1 virus-infected A549 cells (Fig. 6b). 5Z added at 12 hpi did not inhibit the NP and NS1 expression (Fig. 6b). IF staining revealed that 5Z restored occludin and claudin-1 expression on the cell membrane of H5N1 virus-infected A549 cells (Fig. 6c). 5Z alone did not change the pattern and levels of occludin and claudin-1 expression in uninfected A549 cells (Fig. 6c). Quantification of immunofluorescence intensity revealed that H5N1 virus infection significantly decreased the occludin and claudin-1 signals located in intercellular junctions, which was largely prevented by 5Z (Fig. 6d). We conducted a TEER assay to determine if H5N1 virus infection led to the increased paracellular permeability of A549 cell monolayer seeded in the Transwell inserts. H5N1 viruses (1 MOI) significantly decreased TEER in a time-dependent manner (Fig. 6e). The addition of 5Z (5 μM) did not alter TEER values of uninfected cells but almost completely restored the electric resistance in H5N1 virus-infected A549 cells (Fig. 6e).

**Fig. 7:**
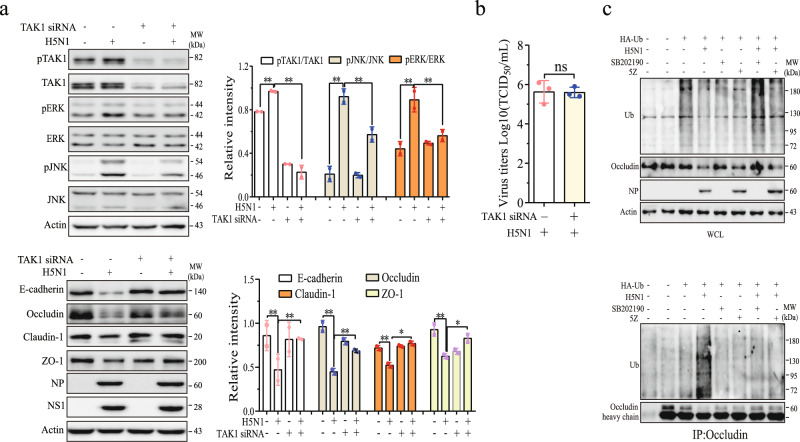
TAK1 siRNA blocks the H5N1 virus-induced degradation of junction proteins. **a** A549 cells were transfected with a scrambled control or TAK1 siRNA. After incubation for 24 h, the cells were left uninfected or infected with H5N1 viruses (1 MOI) and incubated for another 24 h. Cell lysates were analyzed for TAK1, ERK, and JNK phosphorylation and for the levels of epithelial junction proteins by Western blot with the indicated antibodies. Relative protein levels were analyzed by quantification of the density of the protein bands with NIH Image-J software and presented as bar graphs. Data are the mean ± SD of three experiments. *p < 0.05, **p < 0.01. **b** The conditioned media from H5N1 virus-infected cells were analyzed for virus titers by measuring the TCID50 values. **c** H5N1 virus-induced occludin ubiquitination is blocked by TAK1 or p38 inhibitors. A549 cells were transiently transfected with the expression vector encoding the HA-ubiquitin gene. After incubation for 24 h, the cells were left uninfected or infected with H5N1 viruses (1 MOI) and then incubated in the absence or presence of 5Z (5 μM) or SB202190 (10 μM). Cell lysates were prepared and immunoprecipitated with an antibody against occludin. Whole-cell lysates (WCL) and immunoprecipitates were analyzed with antibodies against ubiquitin, NP, occludin, and β-actin. The data represent one of three independent experiments.

We next determined whether TAK1 knockdown also blocked H5N1 virus-induced downregulation of intercellular junction proteins. As shown in Fig. 7a, TAK1 expression was almost completely silenced by TAK1 siRNA in uninfected and H5N1 virus-infected A549 cells. TAK1 siRNA blocked ERK and JNK phosphorylation induced by H5N1 viruses (Fig. 7a). TAK1 silencing alone did not significantly increase E-cadherin, occludin, claudin-1, and ZO-1 expression in uninfected A549 cells but largely restored the levels of these proteins in H5N1 virus-infected cells (Fig. 7a). TAK1 silencing did not alter NP and NS1 protein expression (Fig. 7a) nor the virus titers in the conditioned media of H5N1 virus-infected cells (Fig. 7b).

We then evaluated the effect of TAK1 and p38 inhibitors on H5N1 virus-induced occludin ubiquitination. As shown in Fig. 7c, H5N1 viruses increased occludin ubiquitination. 5Z and SB202190 blocked H5N1 virus-induced occludin ubiquitination. These results suggest that H5N1 viruses downregulate occludin by TAK1-mediated Itch induction and subsequent occludin ubiquitination.

### H5N1 viruses downregulate the levels of intercellular junction proteins in the lung tissue of mice

**Fig. 8:**
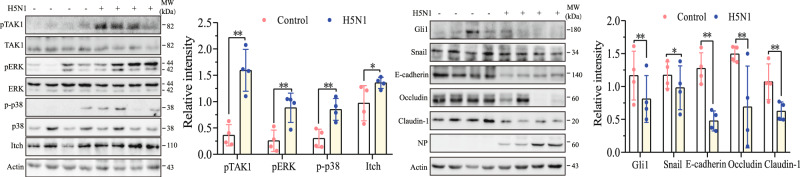
IAV infection activates the TAK1-Itch axis and decreases the levels of epithelial junction proteins in vivo. C57BL/6 mice were left uninfected or infected with H5N1 viruses (1000 pfu/mouse, 4 mice/group). Forty-eight hours post-infection, mice were sacrificed. The lung tissue was collected and analyzed for TAK1, p38, and ERK phosphorylation and the levels of Itch and the epithelial junction proteins by Western blot with the indicated antibody. The density of the bands from four mice per group was analyzed by using NIH Image-J software and normalized by the arbitrary units of β-actin. Data represent the mean ± SD of three experiments. *p < 0.05, **p < 0.01.

**Fig. 9:**
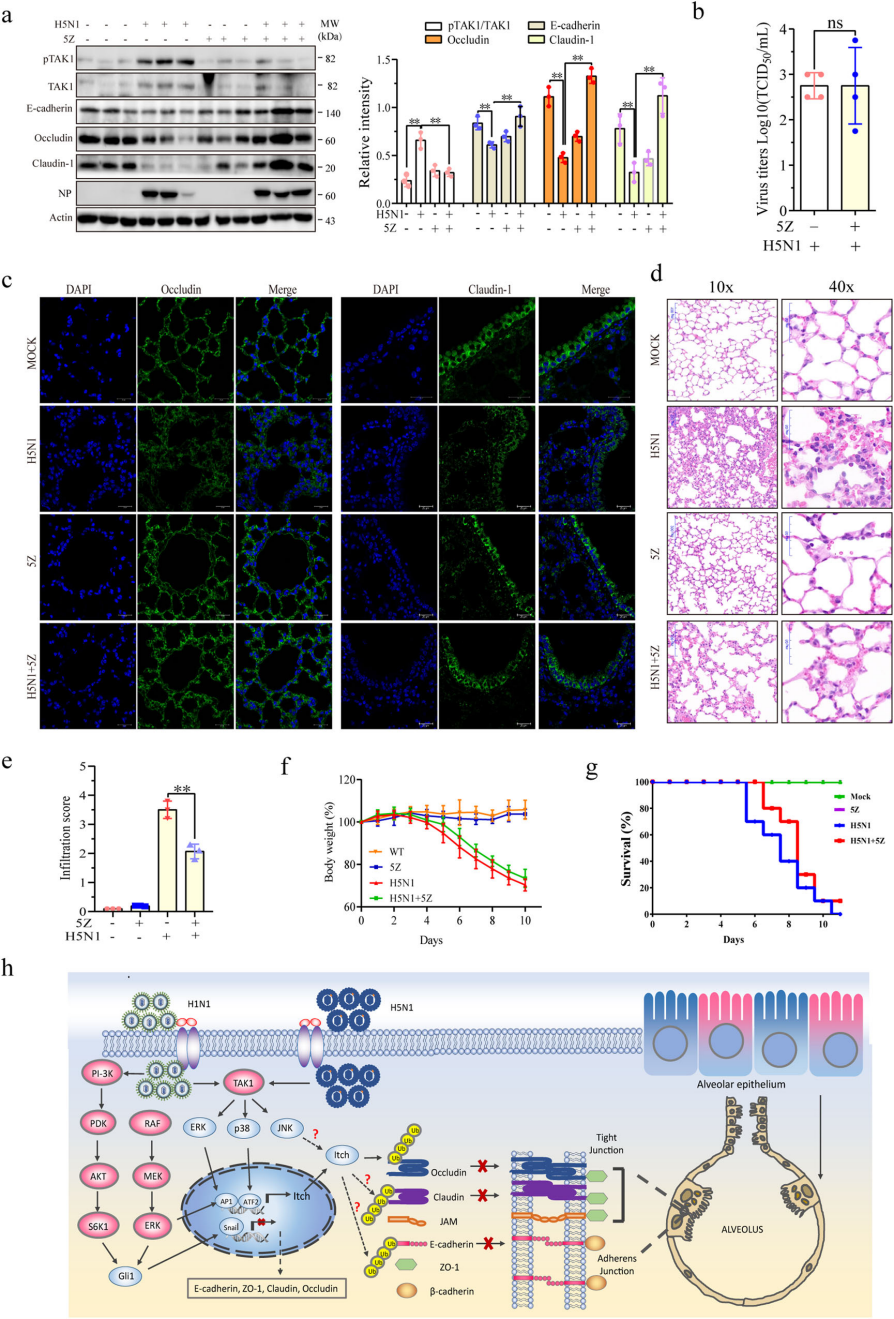
5Z suppresses IAV-induced TAK1 phosphorylation and restores the levels of epithelial junction proteins in vivo. **a** 5Z inhibits H5N1 virus-induced downregulation of intercellular junction proteins. C57BL/6 mice were first treated with vehicle or 5Z (2 mg/kg body weight) 12 hr before virus infection. Mice were left uninfected or infected with H5N1 viruses (1000 pfu/mouse, 4 mice/group) and then treated daily with the same dose of vehicle or 5Z. Mice were sacrificed 48 hr after virus infection. One portion of lung tissue was analyzed for the levels of TAK1, Snail, and junction proteins by Western blot with the indicated antibodies. The density of the bands from 8 mice per group was analyzed using NIH Image-J software and normalized by the arbitrary units of β-actin. *p < 0.05, **p < 0.01. **b** 5Z does not affect H5N1 virus replication. **c** 5Z blocks H5N1 virus-damaged intercellular junction structure. The sections of the paraffin-embedded lung tissue blocks were stained with antibodies against occludin and claudin-1 as described in Methods. **d**, **e** 5Z improves the pathological changes in the lungs of H5N1 virus-infected mice. Sections of paraffin-embedded lung tissue blocks were stained with H & E **d** and graded based on the filtration of inflammatory cells as described in Methods. Data represent the mean ± SD from three sections per mouse, 4 mice per group **e**. *p < 0.05, **p < 0.01. **f**, **g** Female C57BL/6 mice (6-8 weeks old, 10 mice/group) were mock-infected with PBS or infected intranasally with H5N1 viruses (1000 pfu/mouse) and then treated daily with the vehicle or 5Z (2 mg/kg body weight) for 7 days. Mice were weighed and monitored for survival for 16 days. Percent of bodyweight changes **f** and percent survival **g** were plotted. *p < 0.05, compared to the untreated controls. **h** Schematic model of H5N1-induced disruption of intercellular junctions. H5N1 virus activates TAK1 through the Toll-like receptor 3 (TRL3), leading to the activation of p38 and ERK kinases. These kinases induce the expression of Itch, which functions as a ubiquitin ligase to induce occludin ubiquitination and degradation of occludin. Whether Itch mediates the degradation of intercellular junction proteins remains to be investigated. In contrast, H5N1 viruses activate the PI-3 kinase pathway but do not activate p38 and JNK in alveolar epithelial cells. Activation of the Gli1 and Snail transcription factors by H5N1 viruses suppresses the expression of intercellular junction proteins. H5N1 virus infection may also downregulate the levels of intercellular junction proteins in part by inducing the proteasomal degradation of these proteins.

Finally, we investigated whether H5N1 virus infection had the same effects in the lungs of virus-infected mice. As shown in Fig. 8, H5N1 virus infection significantly increased Itch and the levels of TAK1, p38, and ERK phosphorylation but decreased the levels of Gli1, Snail, E-cadherin, occludin, and claudin-1 in the lung tissue of H5N1 virus-infected mice. Of note, the levels of protein phosphorylation and total proteins among individual mice appear to be variable. This is probably due to the inherited nature of in vivo samples. We next determined the in vivo effect of 5Z on the expression of TAK1, Snail, and intercellular junction proteins in IAV-infected mice. 5Z treatment blocked H5N1 virus-induced TAK1 phosphorylation in the lung tissue and restored the expression of E-cadherin, occludin, and claudin-1 (Fig. 9a). 5Z treatment did not affect the levels of the NP protein of H5N1 viruses in the lung tissue (Fig. 9a). IF staining revealed that occludin and claudin-1 expression was significantly decreased in the intercellular junctions of alveolar and bronchial epithelial cells, respectively, in the lungs of H5N1 virus-infected mice (Fig. 9b). 5Z treatment restored the expression of occludin and claudin-1 in the lung tissue of H5N1 virus-infected mice. 5Z treatment did not significantly change the virus loads in the lung tissue (Fig. 9c). H&E staining revealed widespread edema and inflammatory cell infiltration in the lung tissue of H5N1 virus-infected mice (Fig. 9d). 5Z treatment blocked these pathological changes in H5N1 virus-infected lungs (Fig. 9d). The pathological scores were significantly higher in the IAV-infected lungs than in the uninfected controls. 5Z treatment decreased the pathological scores of the IAV-infected lungs, compared to the untreated controls (Fig. 9e). Finally, we investigated if 5Z treatment had any therapeutic effect. As shown in Fig. 9f, 5Z treatment did not change the bodyweights of H5N1 virus-infected mice, compared to the vehicle controls. 5Z treatment also did not affect the bodyweights of uninfected mice. 5Z treatment did not significantly prolong the survival of H5N1 virus-infected mice (Fig. 9g).

## Discussion

IAV infection impairs the alveolar barrier by disrupting the intercellular junction structure^1,39^. We recently reported that the H1N1 virus increases the paracellular permeability of the alveolar epithelium in part by activating the sonic hedgehog signaling pathway to downregulate the expression of intercellular junction proteins^32^. Our present study demonstrates that the H5N1 virus utilized a different strategy to downregulate the expression of intercellular junction proteins. Specifically, our study reveals that H5N1 virus infection activated TAK1 and two downstream MAP kinases, p38 and ERK. Activation of p38 and ERK leads to increased expression of Itch, an E3 ubiquitin ligase that ubiquitinated occludin and potentially other intercellular junction proteins^7,8^. Ubiquitinated junction proteins were rapidly degraded through the proteasomal pathway. In vivo experiments revealed that H5N1 virus infection induces TAK1 and p38 phosphorylation and increased Itch levels but downregulated intercellular junction proteins in the lung of H5N1 virus-infected mice. Our study highlights the importance of posttranslational regulation of intercellular junction proteins in mediating IAV-induced disruption of the alveolar epithelial barrier.

It has been long recognized that the expression of intercellular junction proteins can be regulated at the transcriptional and posttranslational levels in cancer cells^7,8,40^. In particular, the Shh pathway can transcriptionally downregulate the expression of intercellular junction proteins such as E-cadherin in a variety of malignancies^41^. Emerging evidence suggests that viral infections can also regulate the expression of intercellular junction proteins by Gli1, a transcription factor in the Shh pathway^42,43^. For example, the hepatitis B (HBV) virus upregulates the expression of Gli1 and its target genes in hepatocytes^44–46^ by stabilizing Gli1 through its X gene^47,48^. Epstein-Barr virus (EBV) and human immunodeficiency virus (HIV) induce Gli1 expression in nasopharyngeal carcinomas and their tumor cell lines^49^ and in kidney tissue and glomerular podocytes^50^, respectively. We and others have shown that the H1N1 virus directly or indirectly activates Gli1 in *Drosophila* and in mammalian cells, leading to the disruption of their intercellular junctions^32,51^. Surprisingly, the H5N1 virus did not increase but rather lowered the levels of Gli1 and Snail proteins. Since both H1N1 and H5N1 viruses increased Gli1 and Snail mRNA levels^32^ (Fig. 1d), we speculate that the decrease of Gli1 and Snail protein levels in H5N1 virus-infected cells is due to their posttranslational regulation. Indeed, both Gli1 and Snail can be ubiquitinated and undergo proteasomal degradation^52,53^. Notably, although H5N1 virus infection decreased the levels of Gli1 protein, the mRNA levels of its downstream target gene, Snail, were increased. This suggests that Snail could be upregulated by other transcription factors such as NF-κB^54^, which was indeed activated by TAK1 in H5N1-virus activated A549 cells (Fig. 6b).

Itch plays an important role in regulating inflammation and the immune response^34,35^. Itch activity is positively regulated by JNK and ATM and negatively regulated by the Fyn tyrosine kinase^52,55,56^. Itch ubiquitinates numerous substrates including Gli1, c-Jun, JunB, SuFu, B-Raf, p73, and p63^52,55,56^. We recently reported that H5N1 but not H1N1 virus activates JNK^33,57^. Interestingly, H1N1 viruses induce Gli1 expression^32^, whereas H5N1 viruses induced Gli1 degradation (Fig. 1a). These observations suggest that, while H5N1 virus only increased the levels of Itch protein weakly or modestly, Itch activity can be further increased by JNK-mediated phosphorylation and ATM activation. Moreover, we found that Itch was required for H5N1 virus-induced ubiquitination and degradation of occludin. This observation is in line with a prior study showing that Itch interacts with occludin and induces its ubiquitination and degradation^58^. VEGF stimulates occludin phosphorylation at serine 490 and promotes Itch-mediated ubiquitination^9^. Since inhibition of Itch expression also blocked H5N1 virus-induced E-Cadherin, claudin-1, and ZO-1, it appears that Itch may also downregulate the expression of these junction proteins as well as Gli1 and Snail by ubiquitination and degradation. It should be noted that in addition to the transcriptional regulation and post-translational modification, the expression of these intercellular proteins could also be controlled at the translational level by accelerating cellular mRNA degradation and the shutoff of protein biosynthesis. It is unlikely that H5N1 virus infection lowered the levels of cellular proteins by inducing cell death since A549 cells do not undergo necroptosis^59^, and inhibition of apoptosis by Z-VAD-FMK (Z-Vad), a pan-caspase inhibitor, did not restore the levels of intercellular proteins (Supplementary Fig. 2). Nevertheless, our study suggests unambiguous evidence that Itch plays a crucial role in downregulating occludin expression.

In addition to suppressing the expression of various cellular proteins, Itch activation by the PI-3 kinase pathway in H1N1 virus-infected A549 cells supports IAV replication^60^. Though H5N1 viruses do not activate the PI-3 kinase pathway (Fig. 9h) but can induce Itch expression and activate it through TAK1. However, Itch knockdown did not change the levels of the viral proteins in H5N1 virus-infected A549 cells (Fig. 3b) nor the virus titers in the conditioned media (Fig. 3c). In contrast, Su et al^60^ reported that Itch inhibition slows down IAV replication. The discrepancy is likely because the dose of H5N1 virus used to infect A549 cells was much higher than that used by Su et al.^60^. In addition, H5N1 virus replicates much faster than H1N1 virus.

The MAP kinase pathway has been implicated in destabilizing tight junction structure by down-regulating the expression of intercellular junction proteins^61^. For example, activation of the ERK kinase pathway in HIV-infected human brain endothelial cells and in Endophilin-1-stimulated endothelial cells results in decreased ZO-1 and occludin expression^62,63^. Our recent study suggests that the H1N1 virus downregulates the expression of intercellular junction proteins in part by ERK-activated Gli1 (Fig. 9h)^32^. Our present study shows that H5N1 viruses induced Gli1 mRNA expression (Fig. 1d). However, Gli1 protein levels in H5N1 virus-infected A549 cells were not correspondingly increased but rather decreased, probably resulting from Itch-mediated ubiquitination and accelerated degradation. Nevertheless, we found that U0126, a MEK inhibitor, was able to block H5N1 virus-induced Itch expression. This suggests that ERK activation by the H5N1 virus contributes to the downregulation of intercellular junction proteins by inducing Itch expression. Along this line, p38, another MAP kinase, was activated by the H5N1 virus. Inhibition of p38 activity by SB202190 blocked H5N1 virus-induced Itch expression and junction protein downregulation. Consistent with this observation, Jia et al.^36^ recently reported that p38 activation is required for Itch upregulation and occludin downregulation in the testis-blood barrier in Sertoli cells treated with the polychlorinated biphenyls mixture aroclor 1254, a group of environmental endocrine-disrupting compounds. Since JNK activates Itch^64,65^, H5N1 but not H1N1 virus activates JNK (Fig. 9h)^33,57^, one would anticipate that JNK activation contributes to Itch activation in H5N1 virus-infected cells. Unexpectedly, we found that SP600125, a JNK inhibitor, did not affect H5N1 virus-induced downregulation of intercellular junction proteins (Fig. 5f). It remains an enigma why inhibition of JNK activity, which should downregulate Itch activity, did not lead to the restoration of intercellular junction proteins (Fig. 9h).

TAK1 is a serine/threonine kinase that can be activated by viral proteins or by Toll-like receptor (TLR) signaling. For example, the Vpr protein and the gp41 glycoprotein of HIV-1 can bind and directly activate TAK1, leading to NF-κB activation and increased virus replication^66,67^. The A52 protein of poxvirus interacts with TRAF6 to indirectly activate TAK1, leading to p38 but not NF-κB activation^68^. The latent membrane protein 1 (LMP1) of the Epstein–Barr virus binds and activates TAK1, leading to JNK activation^69^. We recently reported that the H5N1 virus activates TAK1, leading to increased JNK phosphorylation and autophagy^57^. Wang et al. recently reported that TLR signaling by poly(I:C) or viral double-stranded RNA induces TAK1^T187/S192^ phosphorylation and its activation^70,71^. Our prior study suggests that the NS1 protein of H5N1 viruses can activate TAK1 by an unknown mechanism^57^. Our additional study herein shows that transfection with an expression vector encoding the NS1 gene of H5N1 virus led to the downregulation of occludin and claudin-1 but not E-cadherin and ZO-1 (Supplementary Fig. 3). Mechanistically, TAK1 activation by the H5N1 virus led to p38, ERK, and JNK activation, and that p38 and ERK kinases were required for the H5N1 virus-induced Itch expression and intercellular junction protein downregulation. These observations collectively suggest that TAK1 activation plays a central role in H5N1 virus-induced downregulation of some intercellular proteins.

TAK1 activates diverse signaling pathways including NF-κB activation, necroptosis, inflammation, innate immunity, and autophagy^37,38^. Our in vivo investigation reveals that 5Z treatment did not affect the levels of viral proteins and virus loads in lung tissues nor alleviated the bodyweight loss and prolonged the survival of H5N1 virus-infected mice. The beneficial effect of 5Z treatment on the integrity of intercellular junctions and alveolar permeability is likely complicated by the anti-inflammatory response of NF-κB inhibition and the pro-inflammatory response of necroptosis. In addition, 5Z treatment may also interfere with innate and adaptive immunity against viral infections. Use of a TAK1 inhibitor as adjuvant antiviral therapy to improve lung functions does not seem appealing.

We are aware that several questions remain unanswered in our current study. The first is the mechanism of H5N1 virus-induced downregulation of E-cadherin and claudin-1. While our study suggests that these two proteins are subject to posttranslational regulation in H5N1 virus-infected cells, and Itch appears to be responsible for H5N1 virus-induced degradation of E-cadherin and claudin-1, definitive evidence is lacking. Overexpression of TAK1 led to decreased levels of occludin and claudin-1 but did not significantly decrease E-cadherin expression; whereas overexpression of Itch only significantly decreased occludin expression. The second unanswered question is if Itch activity in H5N1 virus-infected cells is regulated by JNK. While several studies have shown that JNK can phosphorylate Itch at T222 and activate it^64,65^, it is not clear why inhibition of JNK activity by SP600125 did not block H5N1 virus-induced occludin degradation in our study; Third, the mechanisms of H5N1 virus-induced Gli1 and Snail downregulation remain unknown. Itch has been shown to ubiquitinate Gli1 in cancer cells^52,53^, whether this is the case in H5N1-infected cells needs to be proved.

In summary, our study provides evidence that the H5N1 virus increases Itch expression in vitro and in the lung tissue of H5N1 virus-infected mice. Itch ubiquitinates occludin and its degradation. Downregulation of these junction proteins leads to the disruption of the intercellular junction structure and increased paracellular permeability in the alveolar epithelial barrier (Fig. 9h). Mechanistic studies revealed that increased Itch expression was mediated through TAK1-activated p38 and ERK kinases. Our study provides insights into the mechanisms of IAV-induced downregulation of alveolar junction proteins and improves our understanding of the pathogenesis of H5N1 virus infections in lung tissue.

## Methods

### Reagents

SB202190 (#154447-36-6) and U0126 (#1173097-76-1) were purchased from Selleck (Shanghai, China). 5Z (#253863-19-3) was purchased from Santa Cruz Biotechnologies, Inc. (San Diego, CA, USA). Antibodies against Gli1 (#2643), Snail (#3895), Itch (#12117), TAK1 (#5206), phospho-TAK1^S412^ (#9339), p38 (#4685), phospho-p38 (#4060), JNK (#9252), phospho-JNK (#4668), ERK (#4695), phospho-ERK (#9101), and claudin-1 (#13255) were purchased from Cell Signaling Technology, Inc. (Danvers, MA, USA). Itch siRNA (#sc-40364) and antibodies against E-cadherin (sc-8246), occludin (sc-133255), ZO-1 (#sc-10804), NP (#sc-52026), NS1 (#sc-130568), β-actin (#sc-47778), and glyceraldehyde 3-phosphate dehydrogenase (GAPDH) were obtained from Santa Cruz Biotechnology Inc. (San Diego, CA, USA). A rabbit mAb against TAK1 (#ab109404) was purchased from Abcam Inc. (Shanghai, China). Alexa Fluor® 488 anti-Rabbit IgG (#AB_2338840) and Alexa Fluor® 488 anti-Mouse IgG (#AB_2338064) were purchased from Jackson ImmunoResearch Laboratories, Inc. (West Grove, PA, USA). TAK1 siRNA (#6317) was purchased from Cell Signaling Technology, Inc. (Danvers, MA, USA). Itch siRNA was purchased from Santa Cruz Biotechnology (Santa Cruz, CA). The expression vector encoding FLAG-occludin (#86042) was purchase from Addgene (Watertown, MA, USA). The HA-ubiquitin gene was cloned in a pcDNA3.1 express vector as reported in our previous publication^72^.

### Cell culture

A549 (a human lung cancer cell line of alveolar epithelial cell origin) (CCR-185), NL20 (a human non-tumoral alveolar epithelial cell line) (CRL-1503), and MDCK (Madin-Darby canine kidney) (CCR-34) cells were purchased from the American Tissue Culture Collection (Manassas, VA). Cells were grown in DMEM containing 10% fetal bovine serum (FBS). All cell lines were tested negative for mycoplasma contamination by PCR. A/mallard/Huadong/S/2005 (SY strain, H5N1), A/Chicken/Jiangsu/k0402/2010 (CK10 strain, H5N1), and A/goose/Guangdong/Y6/2015 (Y6 strain, H5N6) have been reported previously^73–75^. These viruses were prepared by inoculating 10-day-old specific-pathogen-free embryonic chicken eggs. The virus titers were determined by a 10-fold serial dilution (10^1^ to 10^9^, and each dilution (10^5^–10^9^) in MDCK. The 50% tissue culture infection dose (TCID_50_/100 μl) values were determined by using the standard Reed and Muench method.

### Animals

Animal use was approved by the Institutional Animal Care and Use Committee of Yangzhou University (Approval number #SYXY-23; date of approval: March 5, 2019) and carried out in accordance with the Guide for the Care and Use of Laboratory Animals by the National Research Council. C57BL/6 mice (female, 6-8 weeks) were purchased from the Laboratory Animal Center at Yangzhou University (Yangzhou, China). 5Z stock was prepared by dissolving it in dimethyl sulfoxide (DMSO) and then diluted in PBS prior to use. Twelve hours prior to drug administration, mice were pre-treated once by i.p. injection of 5Z at the dose of 2 mg/kg body weight. Control mice were treated with the vehicle. Mice were then deprived of water 4 h before virus infection. Mice were anesthetized with an intraperitoneal injection of sodium pentobarbital administration (100 mg/kg body weight) and then infected with H5N1 virus (1000 PFU/mouse in 50 μl saline) intranasally. Mice were treated with the vehicle or the same dose of 5Z for two days. Forty-eight hours after virus infection, mice were sacrificed by cervical dislocation. One part of the lung tissue was lysed in NP-40 lysis buffer (weight/volume, 1:50) and analyzed for the indicated proteins by Western blot. A second part of the lung tissue was fixed in 4% paraformaldehyde in PBS and embedded in paraffin within 48 h after fixation. The sections of paraffin-embedded tissue blocks were dehydrated and rehydrated as described previously^76^ and then stained with anti-occludin (mouse mAb) and claudin-1 (rabbit mAb), followed by staining with Alex488-conjugated anti-mouse and anti-rabbit IgG (1:100), respectively, for 1 h at room temperature. Normal rabbit and mouse IgG were used as the negative controls. Finally, the sections were stained with 10 μL DAPI (4’,6-diamidino-2-phenylindole) for 5 min. After washing the cells in PBS, fluorescent images were captured under a Nikon fluorescence microscope.

To determine the therapeutic effect of 5Z, mice (6-8-wks-old, 9-11 mice per group) infected with H5N1 virus as above were treated daily with 5Z (2 mg/kg body weight in 100 μL PBS) by intraperitoneal injection for 7 days. Mice were monitored daily for body weights and survival for 18 days and were humanely sacrificed by CO2 inhalation when they became moribund or when the loss of body weight decreased by > 30%.

### Histopathology

The sections (5 µm) of lung tissue blocks were stained with hematoxylin and eosin (H&E). Pathologic lesions were graded by using a semi-quantitative histology scoring system in a blinded fashion as previously described^77,78^. The severity of lesions was graded on a scale of 0 to 4 (0 for none or very minor, 1 for mild, 2 for intermediate, 3 for moderately severe, and 4 for severe and widespread) based on the following criteria: (1) bronchiolitis (integrity of airway epithelium, necrotic bodies or denudation of airway epithelial lining); (2) inflammation (infiltration of macrophages, neutrophils and lymphocytes): (3) alveolitis (damaged alveolar epithelial cells or denuded epithelial lining); (4) interstitial inflammation (inflammation in the alveoli or thickening of the alveolar interstitium); (5) hemorrhage (presence of erythrocytes in the alveolar air space, damaged capillaries and hemorrhagic effusions in the damaged areas); (6) edema (presence of proteinaceous material in the alveolar air space).

### Real-time quantitative PCR analysis

Total RNA was extracted from A549 cells using TRIzol reagent (Invitrogen, Grand Island, NY, USA). RNA integrity was verified by electrophoresis. Reverse transcription of RNA was performed using the iScript cDNA Synthesis Kit (Bio-Rad, Hercules, CA, USA) according to the manufacturer’s protocol. The cDNA was subjected to quantitative real-time PCR using a PrimeScript RT reagent kit and SYBR Green Supermix (Takara, Dalian, China). The sequences of primers for Gli1: 5′-TTCCTACCAGAGTCCCAAGT-3′, 5′-CCCTATGTGAAGCCCTATTT-3′; Snail: 5′-GGACCCACACTGGCGAGAAG-3′, 5′-ATTCGGGAGAAGGTCCGAGC-3′; E-Cadherin: 5′-CGGGAATGCAGTTGAGGATC-3′, 5′-AGGATGGTGTAAGCGATGGC-3′; Occludin: 5′-CCCTTTTAGGAGGTAGTGTAGGC-3′, 5′-CCGTAGCCATAGCCATAACCA-3′; Claudin-1: 5′-GTGGAGGATTTACTCCTATGCCG-3′, 5′-ATCAAGGCACGGGTTGCTT-3′. Amplification of human GAPDH was included as a control. All expression levels were normalized to GAPDH levels in the same sample. Fold change was calculated by the ΔΔCT method. Percent expression was calculated as the ratio of the normalized value of each sample to that of the corresponding untreated control sample. All Real-Time RT-PCR analyses were performed in triplicate.

### Western blot

Cells grown in 6-well plates were harvested and lysed in NP-40 lysis buffer (50 mM Tris-HCl (pH 8.0), 150 mM NaCl, 1% NP-40, 5 mM EDTA, 10 µg/ml aprotinin, 10 µg/ml leupeptin, and 1 mM phenylmethylsulphonyl fluoride). After incubation on ice for 30 min, the cell lysates were prepared by spinning down at 4 °C, 15,000 rpm for 15 min. Cell lysates were analyzed by Western blot with antibodies (1:1000 or 1:2000) against the proteins of interest, followed by horseradish peroxidase-conjugated goat anti-rabbit or anti-mouse IgG (1:5000) and SuperSignal Western Pico enhanced chemiluminescence substrate (Pierce Chemical Co., Rockford, IL). The density of the bands was analyzed by using NIH Image-J software (NIH, Bethesda, MD, USA) (https://imagej.nih.gov/ij/) and normalized by the arbitrary units of their corresponding total proteins or β-actin as indicated. Quantified results were presented as the mean ± standard deviation (SD) from three experiments in bar graphs.

### Gene silencing

A549 cells seeded in a 6-well plate were transfected with siRNA using Lipofectamine RNAiMAX (Invitrogen Life Technologies, Grand Island, NY) according to the manufacturer’s instruction. After incubation for 48 h, the cells were left uninfected or infected with the H5N1 virus and incubated for 24 h. Cells were harvested and analyzed for the expression of TAK1, Itch, and other relevant proteins by Western blot. The conditioned media from H5N1 virus-infected cells were analyzed for virus titers by measuring the TCID_50_ values.

### Immunofluorescence staining

A549 cells were seeded on coverslips in 24-well flat-bottomed plates and treated with H5N1 *virus* or pretreated with 5Z (5 µM) for 30 min followed by infection with H5N1 virus for 12 h. The cells were fixed with 100% methanol at -20 °C for 20 min. The cells were permeabilized with 0.25% Triton X-100 in PBS for 20 min and washed three times with PBS. The fixed cells were incubated with 5% bovine serum albumin (BSA) blocking buffer in PBS for 30 min at room temperature, and then with anti-occludin and anti-claudin-1 antibodies (1:100) in PBS overnight at 4 °C. Following incubation, the cells were washed in blocking buffer and stained with Alexa488-conjugated anti-mouse and anti-rabbit IgG (1:100), respectively, for 1 h at room temperature. Finally, the cells were stained with 10 μΜ DAPI (4’,6-diamidino-2-phenylindole) for 5 min. After washing the cells in PBS, fluorescent images were captured under a Nikon fluorescence microscope.

### Transepithelial electrical resistance (TEER)

A549 monolayer grown in Transwell filter units (3-μm-diameter pores, Corning, New York, NY, USA) were left uninfected or infected with 1 MOI of H5N1 virus and then incubated in the absence or presence of the indicated inhibitors. The monolayers were analyzed for transepithelial resistance (Epithelial Volt-‘Ohm Meter, Millipore, St. Louis, MO) at the indicated time after virus infection. The measured values were calculated by multiplying the measured electrical resistance by the area of the filter. The results represent the mean ± SD of three independent experiments.

### Statistics and reproducibility

The differences in mRNA levels and Western blot band densities in the cells left uninfected or infected with H5N1 virus in the absence or presence of various treatments were statistically analyzed by using an unpaired Student *t* test. Statistical analyses were carried out based on the data that represent the mean ± SD of three independent experiments. The differences in the half-life of intercellular junction proteins and in TEER values between the uninfected A549 cells and H5N1 virus-infected A549 cells in the absence or presence of the indicated inhibitors were statistically analyzed by using a one-way ANOVA. Data represent the mean ± SD from three independent experiments. The differences in the infiltration scores in the lungs of H5N1 virus-infected mice (*n* = 4) left untreated or treated with 5Z were analyzed by using an unpaired Student *t* test. Differences in the bodyweights of untreated and 5Z-treated mice were analyzed using a repeated measures ANOVA test (*n* = 10). Differences in the survival of untreated and 5Z-treated mice were statistically analyzed by using a Log-Rank test (*n* = 10). A *p* value of < 0.05 was considered statistically significant. All statistics were performed with GraphPad Prism (GraphPad software 8.0 (https://www.graphpad.com/scientific-software/prism).

### Reporting summary

Further information on research design is available in the Nature Research Reporting Summary linked to this article.

## Supplementary information

Supplementary information

Description of Additional Supplementary Files

Supplementary Data 1

Reporting Summary

## Data Availability

The authors declare that the data supporting the findings of this study are available from the corresponding author upon request. Uncropped Western blot images are provided in Supplementary Fig. 4. All relevant data including the numerical and statistical source data that underlie the graphs in figures are provided in Supplementary Data 1.
