# Supplementary material for: H5N1 infection impairs the alveolar epithelial barrier through intercellular junction proteins via Itch-mediated proteasomal degradation
Source: Commun Biol. 2022 Mar 1;5:186. doi: 10.1038/s42003-022-03131-3 (PMC8888635; doi:10.1038/s42003-022-03131-3)
Supplement: Supplementary file 1 — Supplementary information [file 42003_2022_3131_MOESM1_ESM.pdf]

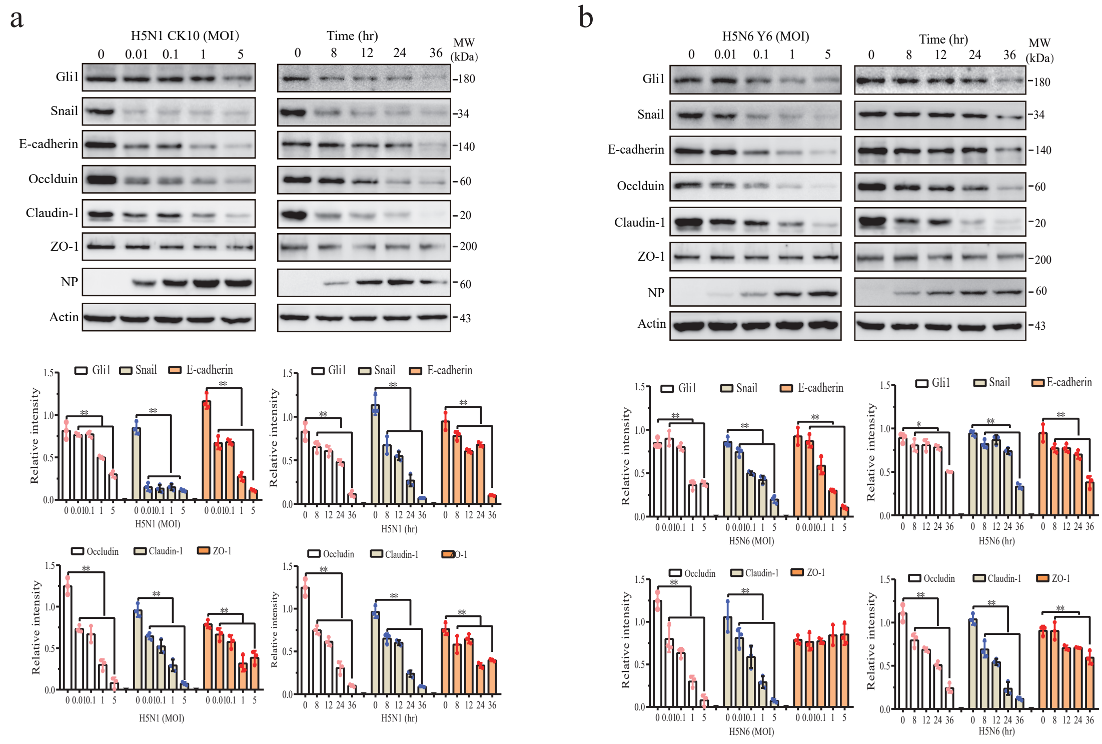

Supplementary figure 1: H5N1 and H5N6 viruses lower the levels of Gli1, Snail, and junction proteins. A549 cells were infected with the various MOI of H5N1 (CK10) (**A**) or H5N6 (Y6) viruses (**B**) for 24 hr or with 1 MOI of viruses for the indicated lengths of time. Cell lysates were analyzed for the expression of Gli1 and Snail and for four junction proteins by Western blot. The density of the bands was analyzed by using NIH Image -J software and normalized by the arbitrary units of β-actin. Data are the mean ± SD of three experiments. *p<0.05, **p<0.01, compared to uninfected control.

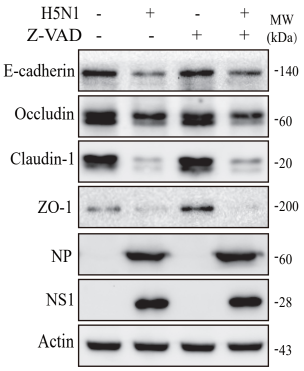

Supplementary figure 2: Apoptosis does not cause downregulation of intercellular proteins. A549 cells were left uninfected or infected with the H5N1 virus (1 MOI). After incubation in the absence or presence of Z-Vad (50 μ M) for 24 hr, cell lysates were prepared and analyzed for the levels of and junction proteins by Western blot with the indicated antibodies. NP, NS1, and Actin were detected as controls.

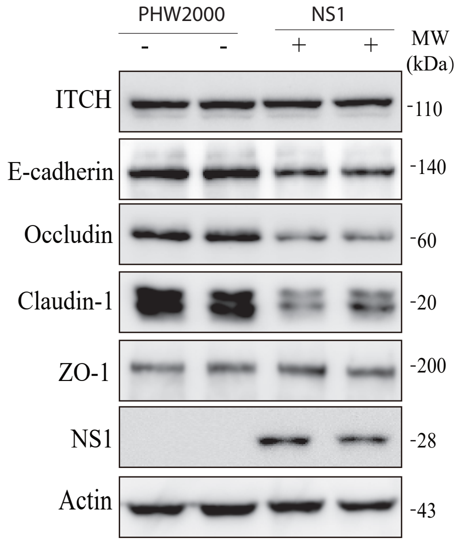

Supplementary figure 3: Downregulation of intercellular proteins by NS1. A549 cells were transfected with PHW2000 or the vector encoding the NS1 gene of the H5N1 virus. After incubation for 48 hr, cell lysates were prepared and analyzed for the levels of junction proteins by Western blot with the indicated antibodies. NS1 and Actin were detected as controls.

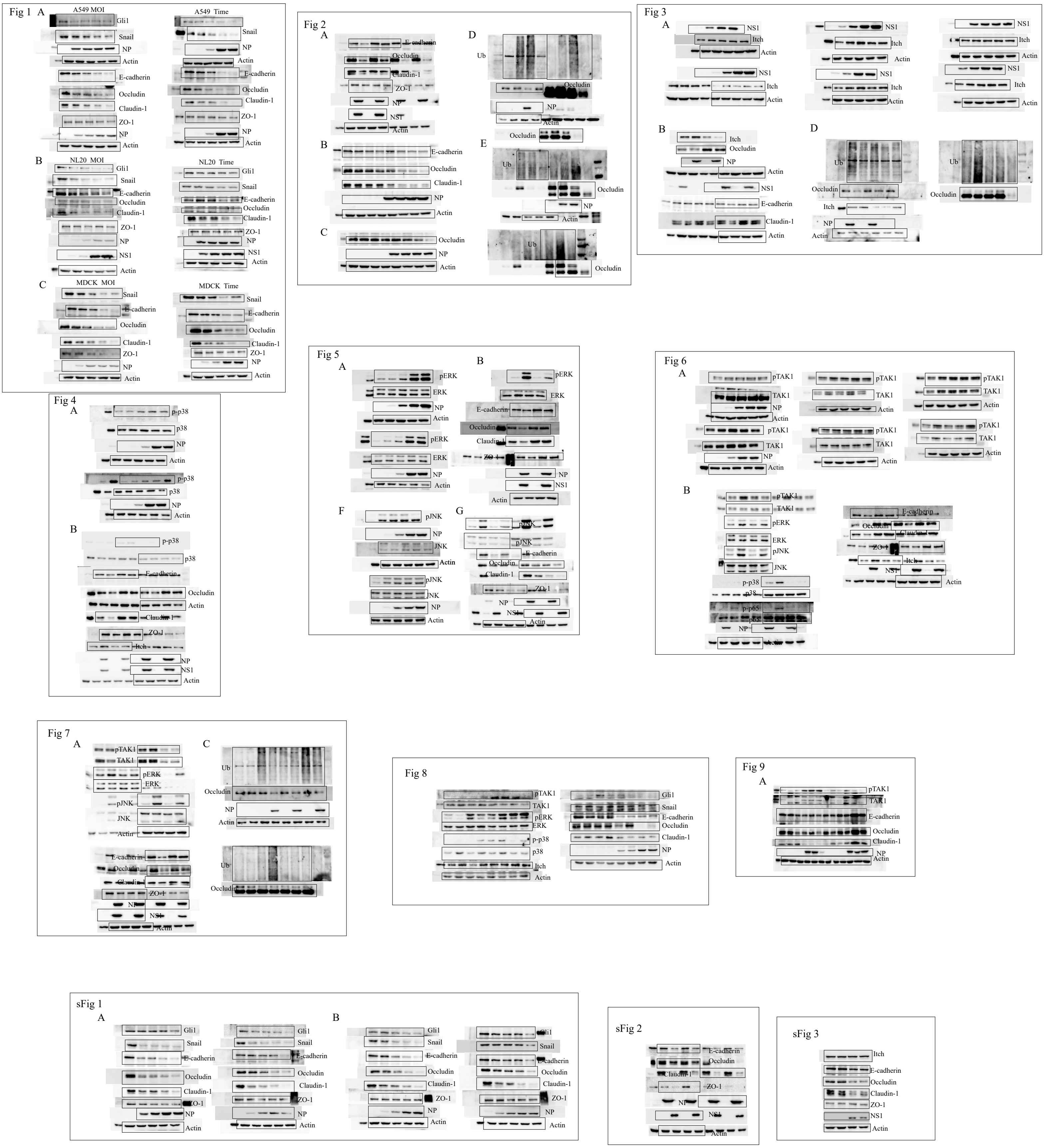

Supplementary figure 4: Images of original Western blots used in figures 1-9 and supplementary figures 1-3.
