# Supplementary material for: H5N1 infection impairs the alveolar epithelial barrier through intercellular junction proteins via Itch-mediated proteasomal degradation
Source: Commun Biol. 2022 Mar 1;5:186. doi: 10.1038/s42003-022-03131-3 (PMC8888635; doi:10.1038/s42003-022-03131-3)
Supplement: Supplementary file 2 — Description of Additional Supplementary Files [file 42003_2022_3131_MOESM2_ESM.pdf]

Description of Additional Supplementary Files

File name: Supplementary Data 1
Description: Source data that underline the graphs in figures.
