# Supplementary material for: H5N1 infection impairs the alveolar epithelial barrier through intercellular junction proteins via Itch-mediated proteasomal degradation
Source: Commun Biol. 2022 Mar 1;5:186. doi: 10.1038/s42003-022-03131-3 (PMC8888635; doi:10.1038/s42003-022-03131-3)
Supplement: Supplementary file 4 — Reporting Summary [file 42003_2022_3131_MOESM4_ESM.pdf]

nature portfolio

Corresponding author(s): Xiulong Xu

Last updated by author(s): December 24, 2021

Reporting Summary

Nature Portfolio wishes to improve the reproducibility of the work that we publish. This form provides structure for consistency and transparency in reporting. For further information on Nature Portfolio policies, see our Editorial Policies and the Editorial Policy Checklist.

Statistics

For all statistical analyses, confirm that the following items are present in the figure legend, table legend, main text, or Methods section.

n/a	Confirmed	
☐	☒	The exact sample size (n) for each experimental group/condition, given as a discrete number and unit of measurement
☐	☒	A statement on whether measurements were taken from distinct samples or whether the same sample was measured repeatedly
☐	☒	The statistical test(s) used AND whether they are one- or two-sided<br>*Only common tests should be described solely by name; describe more complex techniques in the Methods section.*
☒	☐	A description of all covariates tested
☒	☐	A description of any assumptions or corrections, such as tests of normality and adjustment for multiple comparisons
☐	☒	A full description of the statistical parameters including central tendency (e.g. means) or other basic estimates (e.g. regression coefficient) AND variation (e.g. standard deviation) or associated estimates of uncertainty (e.g. confidence intervals)
☒	☐	For null hypothesis testing, the test statistic (e.g. F, t, r) with confidence intervals, effect sizes, degrees of freedom and P value noted<br>*Give P values as exact values whenever suitable.*
☒	☐	For Bayesian analysis, information on the choice of priors and Markov chain Monte Carlo settings
☒	☐	For hierarchical and complex designs, identification of the appropriate level for tests and full reporting of outcomes
☒	☐	Estimates of effect sizes (e.g. Cohen's d, Pearson's r), indicating how they were calculated

Our web collection on statistics for biologists contains articles on many of the points above.

Software and code

Policy information about availability of computer code

Data collection	Images for Western blots were collected and analyzed by using a built-in software from Tanon 5200 Imaging system
Data analysis	The density of Western blots and fluorescence staining was analyzed by using NIH Image-J software (NIH, Bethesda, MD, USA) (https://imagej.nih.gov/ij)

For manuscripts utilizing custom algorithms or software that are central to the research but not yet described in published literature, software must be made available to editors and reviewers. We strongly encourage code deposition in a community repository (e.g. GitHub). See the Nature Portfolio guidelines for submitting code & software for further information.

Data

Policy information about availability of data

All manuscripts must include a data availability statement. This statement should provide the following information, where applicable:

- Accession codes, unique identifiers, or web links for publicly available datasets
- A description of any restrictions on data availability
- For clinical datasets or third party data, please ensure that the statement adheres to our policy

The authors declare that the data supporting the findings of this study are available within the article and the supplementary figures, and from the corresponding authors upon request.

Field-specific reporting

Please select the one below that is the best fit for your research. If you are not sure, read the appropriate sections before making your selection.

☒ Life sciences ☐ Behavioural & social sciences ☐ Ecological, evolutionary & environmental sciences

For a reference copy of the document with all sections, see nature.com/documents/nr-reporting-summary-flat.pdf

Life sciences study design

All studies must disclose on these points even when the disclosure is negative.

Sample size	No sample-size calculation was performed. Four animals per group were used for investigating the impact of H5N1 virus on virus replication, 10 animals per group were used for determining the impact of 5Z treatment on bodyweight and survival. Each in vitro experiment was repeated at least 3 times.
Data exclusions	No data were excluded from the analyses.
Replication	All in vitro experiments were repeated at least three times.
Randomization	Mice were randomly divided into mock-infected or IAV-infected gourp, and then randomly assigned into the untreated or 5Z-treated group.
Blinding	Inflammation scoring in H&E staining, intercellular protein fluorescence staining, and TEER were analyzed by sealing the identification of the samples.

Reporting for specific materials, systems and methods

We require information from authors about some types of materials, experimental systems and methods used in many studies. Here, indicate whether each material, system or method listed is relevant to your study. If you are not sure if a list item applies to your research, read the appropriate section before selecting a response.

Materials & experimental systems

n/a	Involved in the study	
☐	☒	Antibodies
☐	☒	Eukaryotic cell lines
☒	☐	Palaeontology and archaeology
☐	☒	Animals and other organisms
☒	☐	Human research participants
☒	☐	Clinical data
☒	☐	Dual use research of concern

Methods

n/a	Involved in the study	
☒	☐	ChIP-seq
☒	☐	Flow cytometry
☒	☐	MRI-based neuroimaging

Antibodies

Antibodies used	Gli1 (#2643), Snail (#3895), Itch (#12117), TAK1 (#5206), phospho-TAK1S412 (#9339), p38 (#4685), phospho-p38 (#4060), JNK (#9252), phospho-JNK (#4668), ERK (#4695), phospho-ERK (#9101), and claudin-1 (#13255) were purchased from Cell Signaling Technology, Inc. (Danvers, MA, USA); E-cadherin (sc-8246), occludin (sc-133255), ZO-1 (#sc-10804), NP (#sc-52026), NS1 (#sc-130568), Actin (#sc-47778), and glyceraldehyde 3-phosphate dehydrogenase (GAPDH) were obtained from Santa Cruz Biotechnology Inc. (San Diego, CA, USA). A rabbit mAb against TAK1 (#ab109404) was purchased from Abcam Inc. (Shanghai, China). Alexa Fluor® 488 anti-Rabbit IgG (#AB_2338840) and Alexa Fluor® 488 anti-Mouse IgG (#AB_2338064) were purchased from Jackson ImmunoResearch Laboratories, Inc. (West Grove, PA, USA).
Validation	Many antibodies (Gli1, Snail, TAK1, Itch) were validated in this lab by gene knockdown or overexpression. Antibodies against viral proteins were validated by virus infection. Other commonly used antibodies against ERK, p38, JNK were validated by suppliers. Antibodies against intercellular proteins were validated by different applications (membrane location, Western blot with anticipated molecular mass).

Eukaryotic cell lines

Policy information about cell lines

Cell line source(s)	A549 (CCR-185), NL20 (CRL-1503), and MDCK (CCR-34) cell line were purchased from the American Tissue Culture Collection (Manassas, VA).
Authentication	All cell lines authenticated by ATCC were used with limited passages since their arrival.

Mycoplasma contamination	All cell lines were checked routinely by PCR and free of mycoplasma contamination
Commonly misidentified lines (See ICLAC register)	N/A

Animals and other organisms

Policy information about studies involving animals; ARRIVE guidelines recommended for reporting animal research

Laboratory animals	Female C57BL/6 mice (6-8 weeks) were used.
Wild animals	NA
Field-collected samples	NA
Ethics oversight	Animal use was approved by the Institutional Animal Care and Use Committee of Yangzhou University (Approval number #SYXY-23; date of approval: March 5, 2019) and carried out in accordance with the Guide for the Care and Use of Laboratory Animals by the National Research Council.

Note that full information on the approval of the study protocol must also be provided in the manuscript.
